# Looking into the puparium: Micro‐CT visualization of the internal morphological changes during metamorphosis of the blow fly, *Calliphora vicina*, with the first quantitative analysis of organ development in cyclorrhaphous dipterans

**DOI:** 10.1002/jmor.20660

**Published:** 2017-02-09

**Authors:** Daniel Martín‐Vega, Thomas J. Simonsen, Martin J. R. Hall

**Affiliations:** ^1^ Department of Life SciencesNatural History MuseumLondonSW7 5BDUnited Kingdom; ^2^ Naturhistorisk Museum AarhusAarhus CDK‐8000Denmark

**Keywords:** insect development, intra‐puparial period, micro‐computed tomography, organ size, pupal stage

## Abstract

Metamorphosis of cyclorrhaphous flies takes place inside a barrel‐like puparium, formed by the shrinking, hardening and darkening of the third‐instar larval cuticle. The opacity of this structure hampers the visualization of the morphological changes occurring inside and therefore a full understanding of the metamorphosis process. Here, we use micro‐computed tomography (micro‐CT) to describe the internal morphological changes that occur during metamorphosis of the blow fly, *Calliphora vicina* Robineau‐Desvoidy 1830 (Diptera: Calliphoridae) at a greater temporal resolution than anything hitherto published. The morphological changes were documented at 10% intervals of the total intra‐puparial period, and down to 2.5% intervals during the first 20% interval, when the most dramatic morphological changes occur. Moreover, the development of an internal gas bubble, which plays an essential role during early metamorphosis, was further investigated with X‐ray images and micro‐CT virtual sections. The origin of this gas bubble has been largely unknown, but micro‐CT virtual sections show that it is connected to one of the main tracheal trunks. Micro‐CT virtual sections also provided enough resolution for determining the completion of the larval‐pupal and pupal‐adult apolyses, thus enabling an accurate timing of the different intra‐puparial life stages. The prepupal, pupal, and pharate adult stages last for 7.5%, 22.5%, and 70% of the total intra‐puparial development, respectively. Furthermore, we provide for the first time quantitative data on the development of two organ systems of the blow fly: the alimentary canal and the indirect flight muscles. There is a significant and negative correlation between the volume of the indirect flight muscles and the pre‐helicoidal region of the midgut during metamorphosis. The latter occupies a large portion of the thorax during the pupal stage but narrows progressively as the indirect flight muscles increase in volume during the development of the pharate adult.

## 1 Introduction

The morphological changes shown by some insects during metamorphosis has always been both puzzling and captivating for scientists (Erezyilmaz, 2006). One of the first naturalists looking in detail at the differences in the extent of metamorphic changes among different insect groups was Swammerdam (1669), who suggested a classification which, with limitations, was the basis for the current distinction between ametabolous (no metamorphosis), hemimetabolous (partial metamorphosis), and holometabolous (complete metamorphosis) insects (Erezyilmaz, 2006). This classification can be nevertheless problematic (Haug, Haug, & Garwood, 2016) but, in general, the immature stages of most holometabolous (=endopterygote) insects radically differ from the adult stage in morphology, behavior, and ecology. The last larval instar of holometabolous insects transforms into an adult through the pupal stage, which undergoes substantial morphological changes through a more or less extensive histolysis of the larval tissues and subsequent histogenesis of the imaginal ones. Swammerdam (1669) distinguished, however, a fourth group differing from other holometabolous insects in that the insect “does not shed the [larval] skin, but acquires the form of a Nymph under it” (pp. 17–18). This group roughly corresponds to the cyclorrhaphous dipterans, where the pupal stage and the following development of the adult certainly take place inside a barrel‐like puparium, formed from the hardening and darkening of the third‐instar larval cuticle (Fraenkel & Bhaskaran, 1973; Martín‐Vega, Hall, & Simonsen, 2016). This feature, found in only a few other insects (e.g., Strepsiptera, some Hemiptera), allows an extensive and complete histolysis of most larval tissues, as the insect lies within the rigid, protective puparium. Cyclorrhaphous flies include the insect model organism *par excellence*, the fruit fly *Drosophila melanogaster* Meigen, so the metamorphosis of this species has received special attention. However, recent studies of morphology have been few and the studies of Robertson (1936) and Bainbridge and Bownes (1981) still stand as two of the most detailed morphological analyses on the metamorphosis of *D. melanogaster*, as research on this topic in recent years has been mostly focussed on molecular aspects (e.g., Thummel, 1996; Takashima, Mkrtchyan, Younossi‐Hartenstein, Merriam, & Hartenstein, 2008; Rewitz, Yamanaka, & O'Connor, 2010; Rifkin, Kim, & White, 2003).

Although far less studied than *D. melanogaster*, the blow fly *Calliphora vicina* Robineau‐Desvoidy (= *C. erythrocephala* (Meigen)) has also been used as a model organism for the morphological study of insect metamorphosis, especially in the late 19th and the 20th centuries (e.g., Bautz, 1971; Lowne, 1892; Pérez, 1910; Pihan, 1968; Possompès, 1953; Wolfe, 1954). *Calliphora vicina* is a widely distributed, synanthropic species of high economical (Aak, Birkemoe, & Leinaas, 2011) and medico‐legal (Donovan, Hall, Turner, & Moncrieff, 2006) importance. Indeed, at the present time, studies into the metamorphosis of *C. vicina* and other blow flies have seen a renewed interest within a forensic context, as they are typically associated with decomposing organic matter. Staging the intra‐puparial period allows the determination of age‐specific morphological landmarks which, when applied to blow fly puparia collected from a forensic scene, can aid minimum post‐mortem interval estimations (Brown, Thorne, & Harvey, 2015; Richards et al., 2012; Zajac & Amendt, 2012). However, despite its biological significance, and its applied importance in the particular case of blow flies, the internal morphological changes taking place during the metamorphosis of cyclorrhaphous flies are still poorly understood. Moreover, as a consequence of the lack of models of morphological changes in cyclorrhaphous fly metamorphosis, there is a frequent confusion of the concepts in the entomological literature, which might lead to significant errors in applied studies (Martín‐Vega et al., 2016).

In recent years, the use of X‐ray micro‐computed tomography (micro‐CT) and computer‐based 3D‐reconstructions in zoological research has revitalized and revolutionized morphological and developmental studies, enabling the acquisition of high quality data of complex internal structures (Lauridsen et al., 2011; Smith et al., 2016). Furthermore, in contrast to histological techniques, the use of micro‐CT does not require an invasive and time‐consuming dissection of the sample (Carbayo & Lenihan, 2016; Simonsen & Kitching, 2014; Smith et al., 2016). Within a forensic context, Richards et al. (2012) demonstrated the potential of micro‐CT for qualitatively describing internal morphological changes during the metamorphosis of *C. vicina* at 25% time intervals of the total intra‐puparial period. Their preliminary results strongly supported the possibility of establishing a more accurate temporal resolution in further studies. Lowe, Garwood, Simonsen, Bradley, and Withers (2013) demonstrated how micro‐CT can be used to yield volume measurements of selected organs and systems for ontogenetic analyses in a study on the metamorphosis of the painted lady butterfly *Vanessa cardui* (L.). Quantitative data are of particular interest in cyclorrhaphous flies as it has been stated that the insect volume is constant inside the rigid puparium during metamorphosis, in spite of the extensive histolysis and histogenesis which are taking place (Possompès, 1953). From pupariation and until the eversion of the head in the phanerocephalic pupal stage, that is, during the period when most larval tissues degenerate, a compensation mechanism for maintaining a constant volume is the development of a gas bubble which progressively increases in size within the apoptotic larval tissues in the abdominal region (Langley & Ely, 1978), although the origin of this bubble remains unclear (Denlinger & Ždárek, 1994). Despite these recent advances, there is still a lack of quantitative data on the rate of development of the organ systems of the adult fly.

The present study builds on the previous work by Richards et al. (2012), using micro‐CT to describe the morphological changes taking place during cyclorrhaphous fly metamorphosis at a greater temporal resolution than anything hitherto published. The qualitative analysis of the internal morphological changes by Richards et al. (2012) was performed at 25% time intervals of the total duration of the intra‐puparial period (i.e., from pupariation to adult emergence). Our aim is to refine the available temporal resolution to 10% time intervals of the total duration of the intra‐puparial period, and down to 2.5% time intervals during the first 20% interval of the intra‐puparial period, that is, the interval of major morphological changes (Martín‐Vega et al., 2016). Moreover, we aim to provide for the first time quantitative data on the development of different organ systems during the intra‐puparial period. We hope that this study will not only lead to a better understanding of the morphological changes behind an evolutionarily critical process but also facilitate comparative studies of metamorphosis among different holometabolous and between holometabolous and non‐holometabolous insect groups.

## 2 Material and methods

### 2.1 Insect culture and sampling

A laboratory colony of *Calliphora vicina* Robineau‐Desvoidy 1830 was established from adults collected using a modified Redtop® fly trap (Miller Methods, Pretoria) in the Wildlife Garden of the Natural History Museum, London. Newly‐emerged adults from the colony were maintained at a controlled room temperature (23°C ± 2°C) and a daylight cycle of 18:6 hr (light:dark), to prevent the experimental population from entering diapause as post‐feeding larvae (Saunders, 1987; Richards et al., 2012). The flies were provided with sugar, milk powder and water *ad libitum* during one week, and then also with 2 ml of pig blood (from pig liver) once daily during the following ten days, as a protein source for egg maturation. The flies were subsequently starved for 4–5 days, to permit adequate time for egg development. Finally, fresh pig liver was provided as oviposition medium.

Once the flies oviposited, the liver with the eggs was transferred to a plastic box (160 × 160 × 86 mm) containing an approximately 3 cm layer of autoclaved soil, and placed into an incubator at a constant temperature (24°C ± 0.8°C) without light, following standard protocol for blow fly rearing (Donovan et al., 2006). The larvae hatching from the eggs were reared in the same incubator and provided with additional small pieces (*c*. 15 × 5 cm) of fresh pig liver as needed. The feeding stage is comprised by three larval instars, each separated from the previous stage by a cuticular moult (Donovan et al., 2006). Once the post‐feeding larvae started to wander from the food, the box was checked every 6 hr and the white prepupae, that is, irreversibly contracted third‐instar larvae (Fraenkel & Bhaskaran, 1973), were placed into separate plastic boxes (120 × 120 × 60 mm) containing an approximately 1.5 cm layer of autoclaved soil and labeled with the pupariation time. A recent experiment showed that *C. vicina* larvae preferred soil as the substrate for pupariation although there were no differences in the total duration of the intra‐puparial period among different substrates (Hartmann, Martín‐Vega, Hall, & Amendt, 2016). Ten puparia were collected at random from the experimental batch at 0, 6, 12, 18, 24, 30, 36, 42, and 48 hr after pupariation, and then every 24 hr until adult emergence. Puparia not collected from the experimental batch emerged successfully as adults within the expected time range and were transferred to the main colony. The collected puparia were killed and fixed in water near boiling temperature for ∼30 s, and subsequently stored in 80% ethanol at 4°C. This fixation and preservation method has been recommended for morphological studies of blow fly puparia, including histological analyses (Brown, Thorne, & Harvey, 2012). Based on data from an unpublished study carried out at the Natural History Museum (Richards et al., unpublished data), the time required from pupariation to adult emergence by *C. vicina* is approximately 240 hr at 24°C. Therefore, collecting puparia every 24 hr allowed for collecting specimens at each of the eleven 10% time intervals (i.e., from 0% corresponding to pupariation at 0 hr to 100% corresponding to adult emergence at 240 hr after pupariation). The entire procedure was replicated three times, using a different incubator each time to avoid potential bias. Using a constant temperature of 24°C ± 0.8°C for rearing the insects enabled comparison with previous studies on different aspects of *C. vicina* metamorphosis using temperatures of 24–25°C (Bautz, 1971; Pihan, 1968; Possompès, 1953; Richards et al., 2012; Wolfe, 1954; Zajac & Amendt, 2012).

### 2.2 Micro‐CT scanning

Five random puparia (*c*. 9.6 × 3.9 mm) from each batch of ten collected as described above were used for micro‐CT scanning (i.e., 5 puparia × 3 replicates = 15 scanned puparia from each sampled time interval). The remaining five puparia of each batch of 10 were kept as a reserve, and some of them used for histological studies later (see below). Each puparium was pierced in three places using an insect pin (in head, thoracic, and abdominal segments) to enhance the penetration of the staining solution. They were stained by immersion in a 0.5 mol l^−1^ iodine solution for two weeks, then washed and stored in 70% ethanol for 24 hr before scanning. For scanning, each puparium was mounted in a plastic drinking straw containing 70% ethanol and sealed with plastic paraffin film. A batch of five puparia (from the same age and replicate) were scanned together in a Nikkon Metrology HMX ST 225 system (exposure: 500 ms; voltage: 110 kV; current: 100 μA). The resulting projections were reconstructed with a voxel size of 9.5 μm in CT‐Pro 2.1 (Nikon Metrology, Tring, UK). Reconstructed slice stacks in the three principal planes (cross, horizontal, and sagittal) were rendered and visualized for each specimen using VG Studio Max 2.2 (Volume Graphics GmbH, Heidelberg, Germany), for a qualitative analysis of the internal morphological changes. Complete virtual slice stacks for each development interval are available on request from the corresponding author. Terminology for the different intra‐puparial events and stages follows Fraenkel and Bhaskaran (1973) and Martín‐Vega et al. (2016).

Subsequently, the stacks from five randomly selected individuals from the 15 scanned for each 10% development interval among the three replicates were loaded into Avizo 9.0 (Visualization Sciences Group, Bordeaux, France), where selected organ systems were segmented for volume measurements. The selected organ systems were two of the largest ones within the body of the blow fly: the adult alimentary canal and the indirect flight muscles. The alimentary canal is one of the organ systems showing the most substantial changes in morphology during metamorphosis and already Lowne (1892) was aware of the importance of studying those changes in detail. Nevertheless, although this topic has been widely approached from a molecular perspective in *D. melanogaster* (e.g., Hakim, Baldwin, & Smagghe, 2010; Lengyel & Iwaki, 2002; Takashima et al., 2008; Takashima, Younossi‐Hartenstein, Ortiz, & Hartenstein, 2011), most morphological studies include only a broad outline of part of the changes in shape (e.g., Pérez, 1910; Robertson, 1936) and no quantitative data are available. On the other hand, Richards et al. (2012) suggested that the development of the indirect flight muscles, from just short fibres in the first quarter of the intra‐puparial period to occupying almost the entire volume of the thorax at the end of the fourth quarter, might be highly age‐informative if a quantitative measure of this organ system could be achieved. Segmentation was performed automatically using the “Magic wand” tool after redefining the grey scale range within each particular region of interest. The segmented volumes were then reviewed slice by slice and completed with manual segmentation where needed. Quantitative data were obtained using the “Material statistics” module. As some sections from the foregut and the helicoidal region of the midgut were difficult to segment accurately due to lack of contrast, particularly in the last development intervals, segmentation and data quantification of the alimentary canal was restricted to two different regions: the pre‐helicoidal region of the midgut and the rectal pouch in the hindgut. Relative volumes of these structures were also calculated as a percentage of the puparial volume (range 563.27–649.52 mm^3^). Volume measurements were transformed logarithmically on both axes and a simple regression was calculated using the least squares method to describe the relationship between cross‐sectional volume data from the indirect flight muscles and the pre‐helicoidal midgut through development. The regression line was therefore defined as the power law equation log(*y*) = *b* + *k* * log(*x*), where *k* is the allometric coeffient.

Additionally, the data from selected samples were loaded into SPIERS 2.20 (Sutton, Garwood, Siveter, & Siveter, 2012), where the entire alimentary canal (i.e., foregut, midgut, and hindgut) was segmented for 3D‐visualization.

### 2.3 Histological studies

Additional puparia were subjected to histological studies to corroborate the observations from the micro‐CT virtual sections, with a special emphasis on the pupal‐adult apolysis event (i.e., the separation of the epidermal cells of the adult from the pupal cuticle). Four of the 15 remaining puparia (i.e., 5 non‐scanned from each replication) were collected randomly for histological studies at 48, 120, 168, and 240 hr after pupariation (i.e., 20%, 50%, 70%, and 100% of the total intra‐puparial period). The puparial case was removed from each specimen, and the insect was cleared with butanol, embedded in paraffin and sectioned in 10 μm thick sections on a Leica Reichert‐Jung 2040 microtome. The resulting sections were stained with Weigert's haematoxylin, bluish erythrosine, phosphomolybdic acid and fast green and mounted on microscope slides in DPX. Photographs were taken using a Leica® DM6000 B microscope.

### 2.4 2D X‐ray study

The development of the internal gas bubble during early metamorphosis was further investigated with 2D X‐ray imaging using the X‐ray beam of the micro‐CT scanner. A new batch of *C. vicina* eggs were reared under the same conditions as described above (constant temperature of 24°C ± 0.8°C, the white prepupa considered time zero). At 0, 3, 4, 6, 13, 18, 24, 25, 26, 27, 28, 29, and 30 hr after pupariation, 9–10 puparia were collected and stuck by double sided adhesive tape to a Petri dish, approximately 2 mm apart and divided between two rows. The Petri dish was then mounted horizontally on a polystyrene foam base, placed in a Nikon Metrology HMX ST 225 micro‐CT scanner and imaged with an X‐ray beam of 110 kv and 203 µA, through a 0.1 mm aluminium filter. Single images were reconstructed from a set of 32 exposures of 0.5 s. Raw composite images were saved as TIFF files and their brightness adjusted using Adobe Photoshop v. CS4 (Adobe Systems). Gas bubbles were considered as prolate ellipsoids for volume calculation (*V* = 4/3 * π * *a* * 2*b*).

## 3 Results

Table 1 shows a summary of the major morphological changes shown by the main structures throughout the intra‐puparial period. Details on the internal morphological changes are chronologically described below and divided in the three developmental stages taking place inside the puparium: prepupa, pupa, and pharate adult. See Fraenkel and Bhaskaran (1973) and Martín‐Vega et al. (2016), for further details on the delimitation of these developmental stages.

**Table 1:** Summary of the major morphological changes on the main structures during the intra‐puparial period of *Calliphora vicina* reared at 24°C, as shown by virtual micro‐CT sections

Hours after pupariation *(% IPP)*Figures	Developmental stage	Head	Foregut	Midgut	Hindgut	Salivary glands	Indirect flight muscles
0 *(0%)* Figure 1a–c	Prepupa	Head not everted.	Larval foregut visible in the anterior body region.	Larval midgut visible as a long coiled tube.	Larval hindgut visible as a long tube.	Larval salivary glands visible in the anterior body region.	Not developed.
24 *(10%)* Figure 3c–f	Cryptocephalic pupa	Head not everted.	Larval foregut in apoptosis, barely discernible.	Adult midgut visible as a closed sack. Larval midgut transformed into the yellow body, inside the adult midgut.	Larval hindgut in apoptosis but still visible in the caudal part of the abdomen.	Larval salivary glands in apoptosis	Not developed.
48 *(20%)* Figures 6a–f and 13a	Phanerocephalic pupa	Head everted and almost filled with fat bodies. Antennae visible. Cornea and optic nerve visible.	Larval foregut in apoptosis, barely discernible.	Adult midgut visible as a closed sack containing the yellow body, occupying the central part of the thorax.	Adult hindgut starts to profilerate on and around the anterior part of the apoptotic larval hindgut. Rectal pouch partially developed in some individuals.	Larval salivary glands in apoptosis, very reduced. Adult salivary glands partially developed, salivary ducts developing throughout thorax.	Histogenesis of indirect flight muscles starts; small fibres of dorso‐ventral and dorsal‐longitudinal muscles visible.
72 *(30%)* Figure 10a–h	Pharate adult	Small ptilinal invagination and fibres of the ptilino‐oesophageal muscle discernible. Ommatidia present.	Adult oesophagus partially developed. Crop duct proliferates; crop located in the anterior region of the thorax.	Adult midgut “bottle‐shaped,” occupying the central part of the thorax and part of the abdomen.	Adult hindgut developed as a continuous tube. Rectal pouch formed but small.	Larval salivary glands no longer visible. Adult salivary glands further develop.	Indirect flight muscles enlarge.
96 *(40%)* Figure 11a,b	Pharate adult	Ptilinal invagination further develops. The dilator muscles of the pharynx and tentorial muscles start to develop.	Adult oesophagus fully developed. Crop duct elongates; crop located in the posterior region of the thorax.	Adult midgut “long‐necked bottle‐shaped,” thoracic portion stretched and abdominal portion expanded, containing the yellow body. Posterior end tubular and helicoidal.	Adult hindgut elongates and develop a left‐right loop. Rectal pouch enlarges.	Adult salivary glands further develop.	Indirect flight muscles enlarge; fibres surrounded by haemocytes and fat bodies.
120 (50%) Figure 11c,d and 13b	Pharate adult	Ptilinal invagination, dilator muscles of the pharynx and tentorial muscles further developed.	Crop duct further elongated; crop located in the anterior region of the abdomen. Crop expands and flattens lateroventrally.	Pre‐helicoidal region of adult midgut becomes tubular. Helicoidal region elongates and develops left‐right loops.	Adult hindgut further elongates and rectal pouch continues to slightly increase in size.	Adult salivary glands fully developed.	Indirect flight muscles further develop.
144 *(60%)* Figure 11e,f	Pharate adult	Ptilinal invagination, dilator muscles of the pharynx and tentorial muscles further develop.	No further changes.	The pre‐helicoidal region of the adult midgut narrows progressively until the adult emergence.	Rectal pouch continues to increase in size.		Indirect flight muscles further develop.
168 *(70%)* Figures 12a,b and 13c	Pharate adult	Ptilinal invagination, dilator muscles of the pharynx and tentorial muscles further develop.		The pre‐helicoidal region of the adult midgut narrows progressively until the adult emergence.	Rectal pouch continues to increase in size.		Indirect flight muscles further develop.
192 *(80%)* Figure 12c,d	Pharate adult	Ptilinal invagination, dilator muscles of the pharynx and tentorial muscles further develop.		The pre‐helicoidal region of the adult midgut narrows progressively until the adult emergence.	Meconium observable in the rectum. Rectal pouch greatly increases in size.		Indirect flight muscles further develop.
216 *(90%)* Figure 12e,f 240 *(100%)* Figures 12g,h and 13d	Pharate adult	Ptilinal invagination fully developed. Dilator muscles of the pharynx and tentorial muscles fully developed. Crystalline cones and photoreceptor cells layers clearly discernible.		The pre‐helicoidal region of the adult midgut continues to narrow. Yellow body fully absorbed (i.e., not observable).	Meconium fills the rectum and a large portion of the rectal pouch. Lumen of the rectal pouch greatly expanded.		Indirect flight muscles fully develop and attach to the thoracic cuticle.

The correspondent percentage of time of the total intra‐puparial period (IPP) is given in brackets after each time.

### 3.1 Prepupa

#### 3.1.1 0% of the total intra‐puparial period

**Figure 1:**
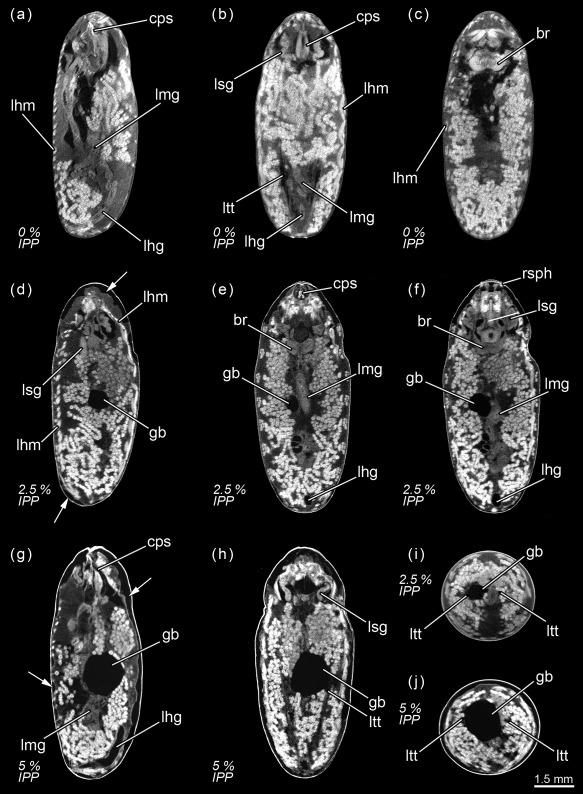
*Calliphora vicina*, micro‐CT‐based virtual sections of puparia at different times after pupariation (AP), reared at 24°C. The corresponding percentage of time of the total intra‐puparial period (IPP) is given in brackets after each time. (**a**) 0 hr AP (0% IPP), medial sagittal section. (**b**) 0 hr AP (0% IPP), dorsal horizontal section. (**c**) 0 hr AP (0% IPP), medial horizontal section. (**d**) 6 hr AP (2.5% IPP), lateral sagittal section. (**e**) 6 hr AP (2.5% IPP), medial horizontal section. (**f**) 6 hr AP (2.5% IPP), ventral horizontal section. (**g**) 12 hr AP (5% IPP), medial sagittal section. (**h**) 12 hr AP (5% IPP), medial horizontal section. (**i**) 6 hr AP (2.5% IPP), medial cross section of the abdomen. (**j**) 12 hr AP (5% IPP), medial cross section of the abdomen. br, brain; cps, cephalopharyngeal skeleton; gb, gas bubble; lfg, larval foregut; lhg, larval hindgut; lhm, larval hypodermal muscles; lmg, larval midgut; ltt, larval tracheal trunks; lsg, larval salivary glands; rsph, respiratory horns. Arrows indicate sites where larval‐pupal apolysis has occurred

Immediately after pupariation, the internal morphology of the white prepupa still resembles that of the third‐instar larva (Figure 1a–c). The living internal tissues are still attached to the larval cuticle (which will harden and darken during the following hours) and the larval hypodermal muscles have not yet started to degenerate (Figure 1a–c). Due to the retraction of the three anterior larval segments (Ždárek & Fraenkel, 1972), the cephalopharyngeal skeleton, the larval salivary glands and the brain are positioned at approximately the same level (Figure 1b–c). Unlike most other larval organs, the brain (Figure 1c) will persist into the adult stage (Hartenstein, 1993). Regrettably, in this and subsequent development intervals, the edges of some neuropils—where neuronal processes contact and form synaptic connections (Ito et al., 2014)—were usually blurry and not well defined. Although iodine staining has been proved to be suitable for analyzing insect neuroanatomy with micro‐CT scanning (Sombke, Lipke, Michalik, Uhl, & Harzsch, 2015), it can result sometimes in more blurred neuropil edges and poorer contrast thresholds in comparison to other staining solutions as phosphotungstic acid (PTA) (Smith et al., 2016). An ongoing study using PTA as the staining method (Smith et al., 2016) is focussing on the reorganization of the brain and eye development during metamorphosis, and therefore few details on these structures will be discussed here.

#### 3.1.2 2.5% Of the total intra‐puparial period

Six hours after pupariation (i.e., 2.5% of the total intra‐puparial period), the larval‐pupal apolysis (i.e., the separation of the epidermal cells of the pupa from the larval cuticle or puparium) is well in progress, albeit at different levels in different body regions: it is nearly complete in the thoracic region, but has only started at some sections of the abdominal region (Figure 1d–h). Contemporaneously to the larval‐pupal apolysis, the extensive histolysis of the larval tissues has started, and the respiratory horns evert and push against the puparial wall (Figure 1f). Also, the scans show a small gas bubble occupying space within the apoptotic larval tissues in the abdominal region (Figure 1d–h), where the larval midgut still has a tubular appearance.

#### 3.1.3 5% Of the total intra‐puparial period

**Figure 2:**
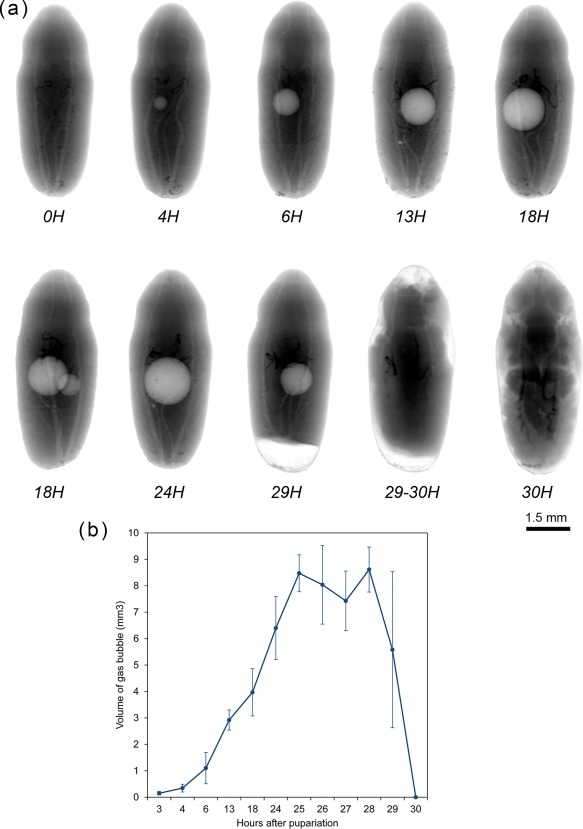
(**a**) *Calliphora vicina*, X‐ray images of puparia in dorsal view, taken at different times after pupariation, reared at 24°C. Times are indicated as hours (H) after pupariation below each imaged specimen. (**b**) average volume ± STD of the gas bubble at different times after pupariation at a constant temperature of 24°C [Color figure can be viewed at wileyonlinelibrary.com]

Twelve hours after pupariation (i.e., 5% of the total intra‐puparial period), the larval‐pupal apolysis is complete in the thoracic region and for the most part of the abdominal region (Figure 1g,h). Furthermore, the gas bubble has increased in volume, whereas the larval midgut appears to have begun to contract (Figure 1g,h). Indeed, X‐ray observations of the gas bubble shows that the bubble is already formed 3–4 hr after pupariation and then progressively increases in volume during the following hours (Figure 2; Table 2). The X‐ray images show how the bubble originates close to one of the main dorsal tracheal trunks; in some cases, two gas bubbles positioned close to each main tracheal trunk can be observed in the same individual (Figure 2a, 18H). Virtual cross sections of 6 and 12 hr‐old prepupae show a connection between the closest tracheal trunk and the gas bubble (Figure 1i,j).

**Table 2:** Volume of the gas bubble at different times after pupariation in *Calliphora vicina* reared at 24°C

Hours after pupariation	Gas bubble volume (mm^3^): average ± STD	Gas bubble volume (mm^3^): range	Number of specimens showing gas bubble
3	0.15 ± 0.07	0.05–0.23	7/10
4	0.34 ± 0.15	0.09–0.51	10/10
6	1.1 ± 0.59	0.42–2.38	9/9
13	2.92 ± 0.38	2.46–3.35	10/10
18	3.97 ± 0.89	2.56–5.28	10/10
24	6.4 ± 1.19	4.64–8.24	10/10
25	8.47 ± 0.7	7.73–9.24	10/10
26	8.04 ± 1.49	5.02–9.7	9/9
27	7.43 ± 1.13	6.44–9.31	8/9
28	8.61 ± 0.85	7.36–9.66	7/10
29	5.58 ± 2.95	2.21–7.72	3/10
30	0	0	0/10

### 3.2 Pupal stage

#### 3.2.1 7.5% of the total intra‐puparial period

**Figure 3:**
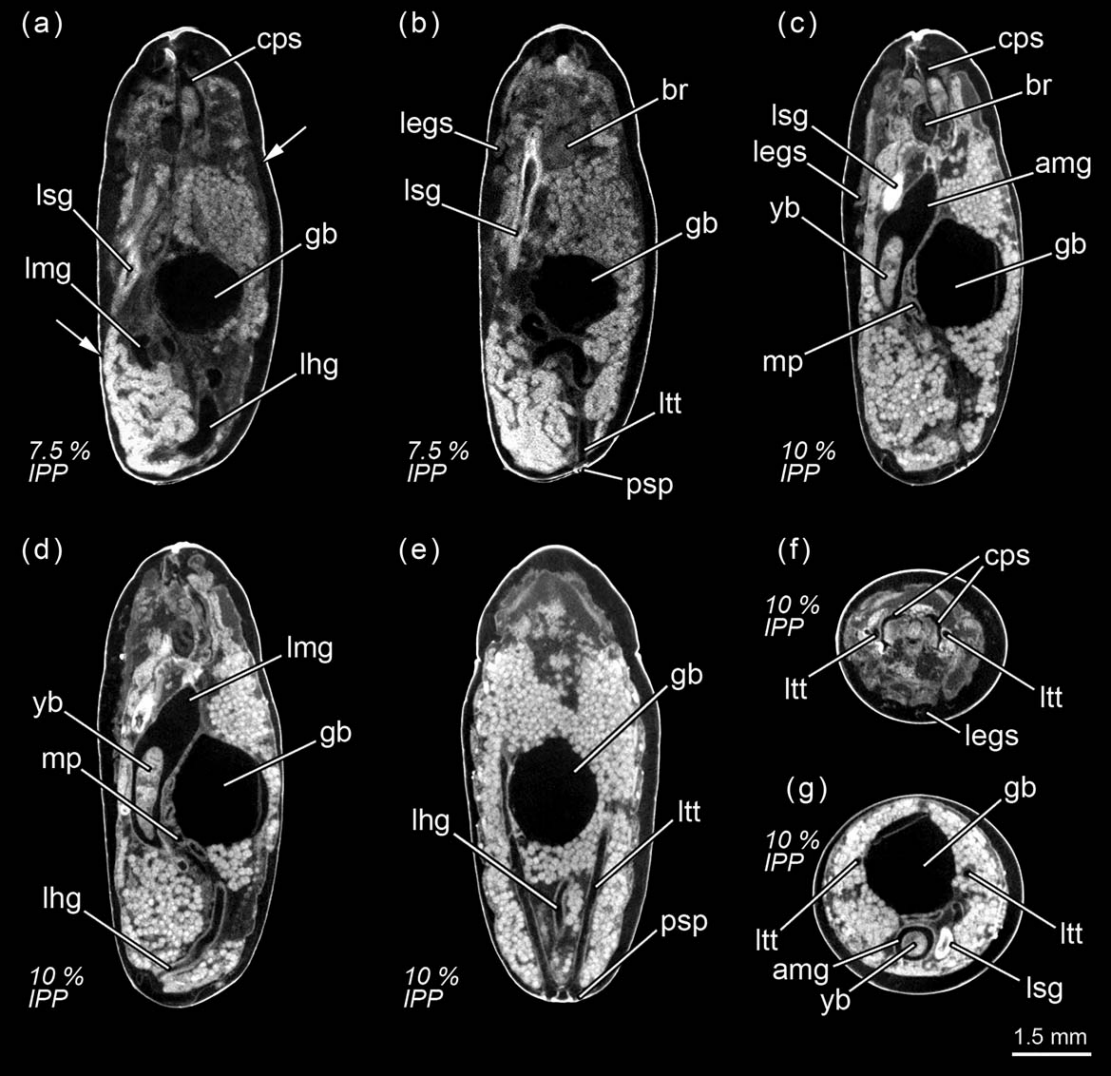
*Calliphora vicina*, micro‐CT‐based virtual sections of puparia at different times after pupariation (AP), reared at 24°C. The correspondent percentage of time of the total intra‐puparial period (IPP) is given in brackets after each time. (**a**) 18 hr AP (7.5% IPP), medial sagittal section. (**b**) 18 hr AP (7.5% IPP), lateral sagittal section. (**c**) 24 hr AP (10% IPP), lateral sagittal section. (**d**) 24 hr AP (10% IPP), medial sagittal section. (**e**) 24 hr AP (10% IPP), dorsal horizontal section. (**f**) 24 hr AP (10% IPP), medial cross section of the thorax. (**g**) 24 hr AP (10% IPP), medial cross section of the abdomen. amg, adult midgut; br, brain; cc, crystalline cones; cps, cephalopharyngeal skeleton; gb, gas bubble; legs, legs; lfg, larval foregut; lhg, larval hindgut; lmg, larval midgut; ltt, larval tracheal trunks; lsg, larval salivary glands; mp, Malpighian tubules; psp, larval posterior spiracles; yb, yellow body. Arrows indicate sites where larval‐pupal apolysis has occurred

Eighteen hours after pupariation (i.e., 7.5% of the total intra‐puparial period), the larval‐pupal apolysis is complete as the epidermis is fully detached from the puparium (Figure 3a,b), although the main tracheal trunks are still attached to the posterior spiracles of the puparium (Figure 3b). At this point, the insect is no longer a prepupa and should be termed a cryptocephalic pupa (Fraenkel & Bhaskaran, 1973; Martín‐Vega et al., 2016). In the cryptocephalic (=“hidden head”) pupa, the cephalopharyngeal skeleton has been partially extruded and the legs and wings have partially everted, but the head remains retracted (Figure 3a,b). The larval midgut continues contracting (Figure 3a).

#### 3.2.2 10% Of the total intra‐puparial period

**Figure 4:**
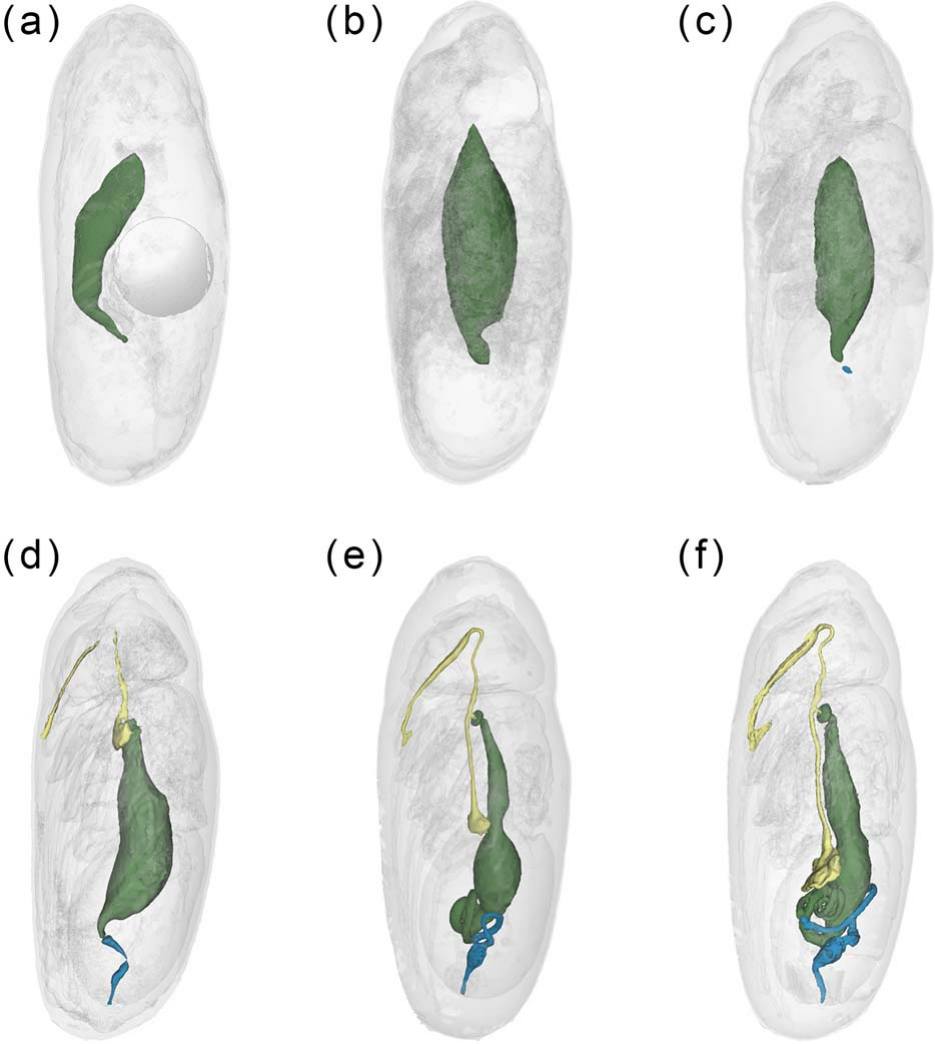
*Calliphora vicina*, false‐color 3D‐surface models of puparia at different times after pupariation (AP), reared at 24°C, showing the changes in the adult alimentary canal. (**a**) 24 hr AP. Note the presence of the gas bubble in the central part of the abdomen. (**b**) 30 hr AP. (**c**) 48 hr AP. (**d**) 72 hr AP. (**e**) 96 hr AP. (**f**) 120 hr AP. Foregut shown in yellow, midgut in green and hindgut in blue

Twenty‐four hours after pupariation (i.e., 10% of the total intra‐puparial period) (Figure 3c–g), the adult midgut has fully contracted, forming a dense mass of apoptotic larval midgut cells, that is, the yellow body, overgrown by the continuous epithelial layer of the adult midgut (Hakim et al., 2010; Takashima et al., 2011). The adult midgut is sack‐shaped, closed at both ends and displaced to the ventral side of the abdomen by the gas bubble (Figures 3c,d,g and 4a), which has continued expanding (Figure 2; Table 2), now occupying the central part of the abdomen (Figure 3g). The Malpighian tubules, which according to Bodenstein (1950) separate from the larval gut and will persist into the adult stage, can be distinguished lying between the bubble and the adult midgut. The apoptotic larval hindgut is still present in the caudal part of the abdomen (Figure 3d,e). The volume of the gas bubble continues to grow until it reaches maximum size; it then remains more or less constant during the following hours. However, 27–30 hr after pupariation the volume rapidly decreases until the bubble disappears as the gas is released into the space caused by apolysis between the pupa and the puparial cuticle (Figure 2; Table 2). The gas from the bubble actually escapes between the pupa and the puparium, at first to the posterior part of the puparium (likely through the closest tracheal trunk) and then surrounding the pupa (Figure 2a). It thus creates the necessary space in the anterior part of the puparium for the head of the pupa to evert by muscular contractions (Hall, Simonsen, & Martín‐Vega, 2017).

#### 3.2.3 12.5% Of the total intra‐puparial period

**Figure 5:**
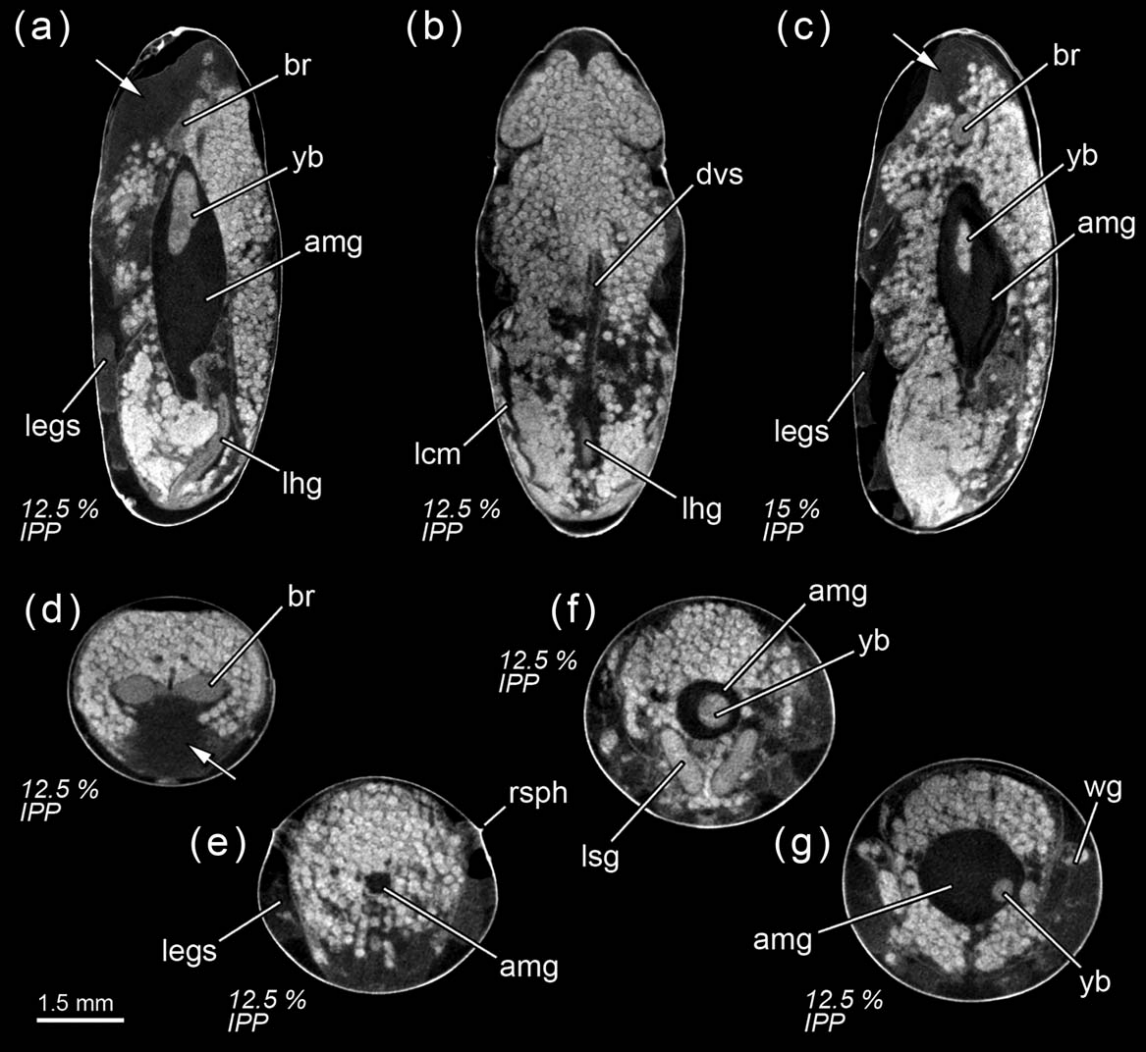
*Calliphora vicina*, micro‐CT‐based virtual sections of puparia at different times after pupariation (AP), reared at 24°C. The corresponding percentage of time of the total intra‐puparial period (IPP) is given in brackets after each time. (**a**) 30 hr AP (12.5% IPP), medial sagittal section. (**b**) 30 hr AP (12.5% IPP), dorsal horizontal section. (**c**) 36 hr AP (15% IPP), medial sagittal section. (**d**) 30 hr AP (12.5% IPP), medial cross section of the head. (**e**) 30 hr AP (12.5% IPP), anterior cross section of the thorax. (**f**) 30 hr AP (12.5% IPP), antero‐medial cross section of the thorax. (**g**) 30 hr AP (12.5% IPP), medial cross section of the thorax. amg, adult midgut; br, brain; legs, legs; lhg, larval hindgut; lhm, larval hypodermal muscles; rsph, respiratory horns; wg, wings; yb, yellow body. Arrows indicate the hyaline regions on the head where the fat bodies have still not migrated

Thirty hours after pupariation (i.e., 12.5% of the total intra‐puparial period), the head, legs and wings have been fully everted (Figures 2a and 5a,b) and, therefore, the cryptocephalic pupa has been transformed into the phanerocephalic (=“visible head”) pupa (Fraenkel & Bhaskaran, 1973; Martín‐Vega et al., 2016). Thus the brain is now located in the head, which is hyaline in appearance until haemocytes and fat bodies migrate from the body and fill it out (Figure 5a–d). As a consequence of the evagination of the head, the respiratory horns (Figure 5e) move backwards and will be projected to the outside of the puparium (see Greenberg, 1991 for more details) through the bubble membrane (Sukontason et al., 2006). Moreover, once the gas bubble has disappeared, the sack‐shaped adult midgut expands and occupies the majority of the thorax and the anterior part of the abdomen (Figures 5a–g and 4b). The abdomen significantly shortens after head eversion and shows an extensive histolysis of remaining larval tissues, such as the abdominal musculature and the hindgut (Figure 5b). No significant changes were observed during the following 6 hr, that is, up until 36 hr after pupariation (i.e., 15% of the total intra‐puparial period), apart from a more advanced histolysis of the larval hindgut (Figure 5c).

#### 3.2.4 17.5–20% Of the total intra‐puparial period

**Figure 6:**
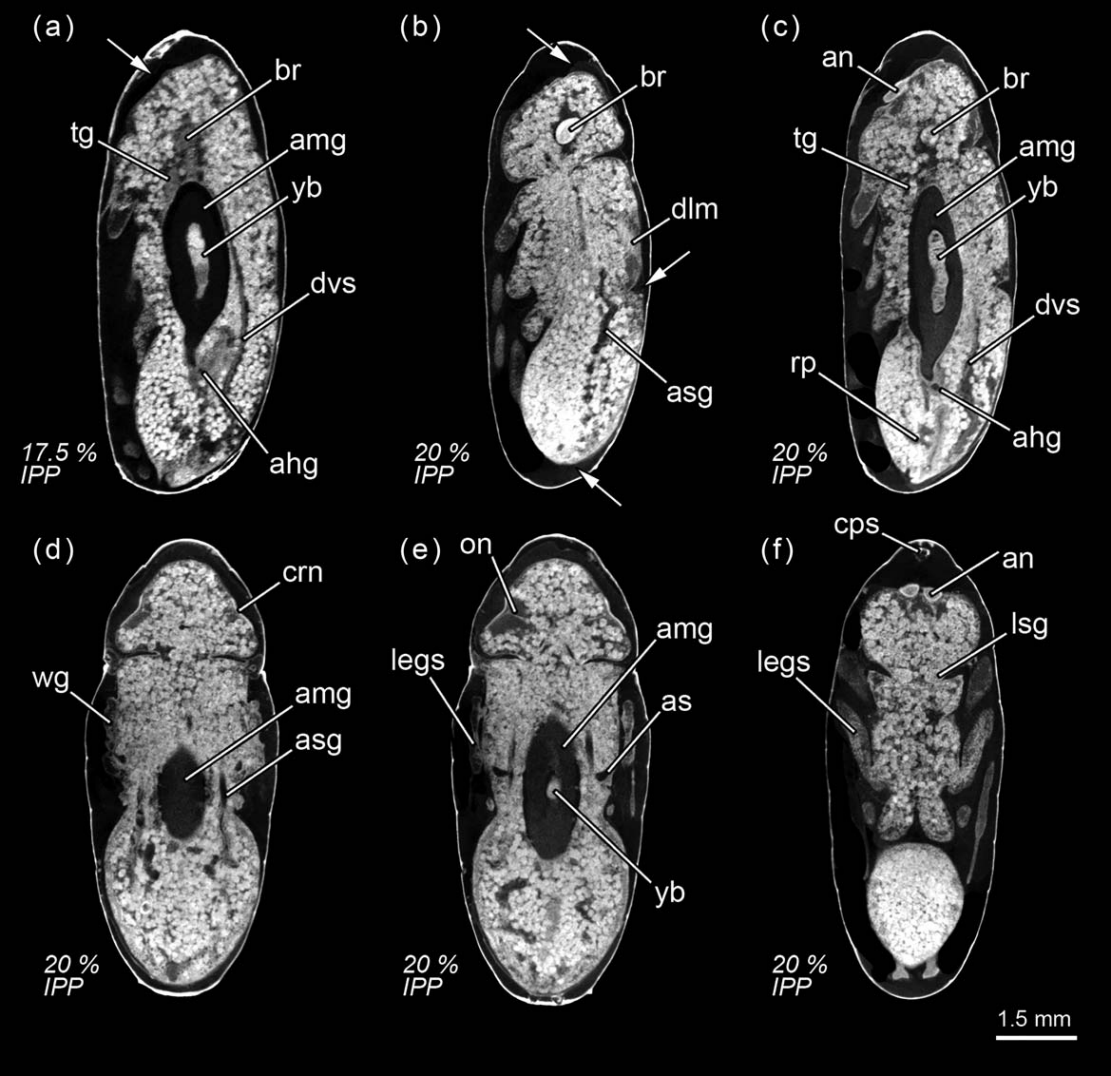
*Calliphora vicina*, micro‐CT‐based virtual sections of puparia at different times after pupariation (AP), reared at 24°C. The corresponding percentage of time of the total intra‐puparial period (IPP) is given in brackets after each time. (**a**) 42 hr AP (17.5% IPP), medial sagittal section. (**b**) 48 hr AP (20% IPP), lateral sagittal section. (**c**) 48 hr AP (20% IPP), latero‐medial sagittal section. (**d**) 48 hr AP (20% IPP), dorsal horizontal section. (**e**) 48 hr AP (20% IPP), medial horizontal section. (**f**) 48 hr AP (20% IPP), ventral horizontal section. ahg, adult hindgut; amg, adult midgut; an, antennae; as, air sac; asg, adult salivary gland; br, brain; cps, cephalopharyngeal skeleton; crn, cornea; dlm, dorsal longitudinal muscles; dvs, dorsal vessel; legs, legs; lsg, larval salivary glands; on, optical nerve; rp, rectal pouch; tg, thoracic ganglion; wg, wings; yb, yellow body. Arrows indicate sites where pupal‐adult apolysis has occurred

**Figure 7:**
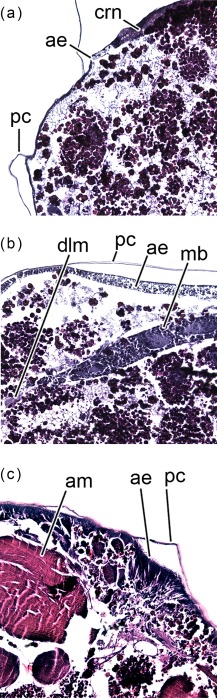
*Calliphora vicina*, histological sagittal sections of puparia reared at 24°C, showing the pupal‐adult apolysis event 48 hr after pupariation. (**a**) head. (**b**) thorax. (**c**) abdomen. ae, adult epidermis; am, abdominal muscles; crn, cornea; dlm, dorsal longitudinal muscles; mb, myoblast; me, meconium; pc, pupal cuticle

**Figure 8:**
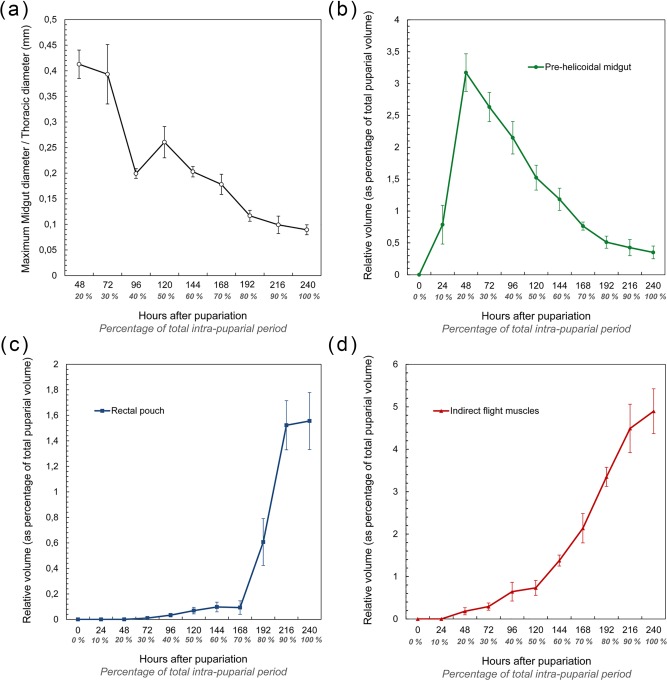
(**a**) average (± STD) relationship between the maximum diameter of the thoracic region of the adult midgut and the maximum diameter of the thorax during the intra‐puparial period. Note that measurements at the 10% development interval are not included as the adult midgut is located in the abdomen at that time. (**b**) average (± STD) relative volume of the pre‐helicoidal region of the adult midgut during the intra‐puparial period. (**c**) average (± STD) relative volume of the rectal pouch of the adult hindgut during the intra‐puparial period. (**d**) average (± STD) relative volume of the indirect flight muscles during the intra‐puparial period. Relative volumes are expressed as a percentage of the total puparial volume (range: 563.27–649.52 mm^3^)

**Figure 9:**
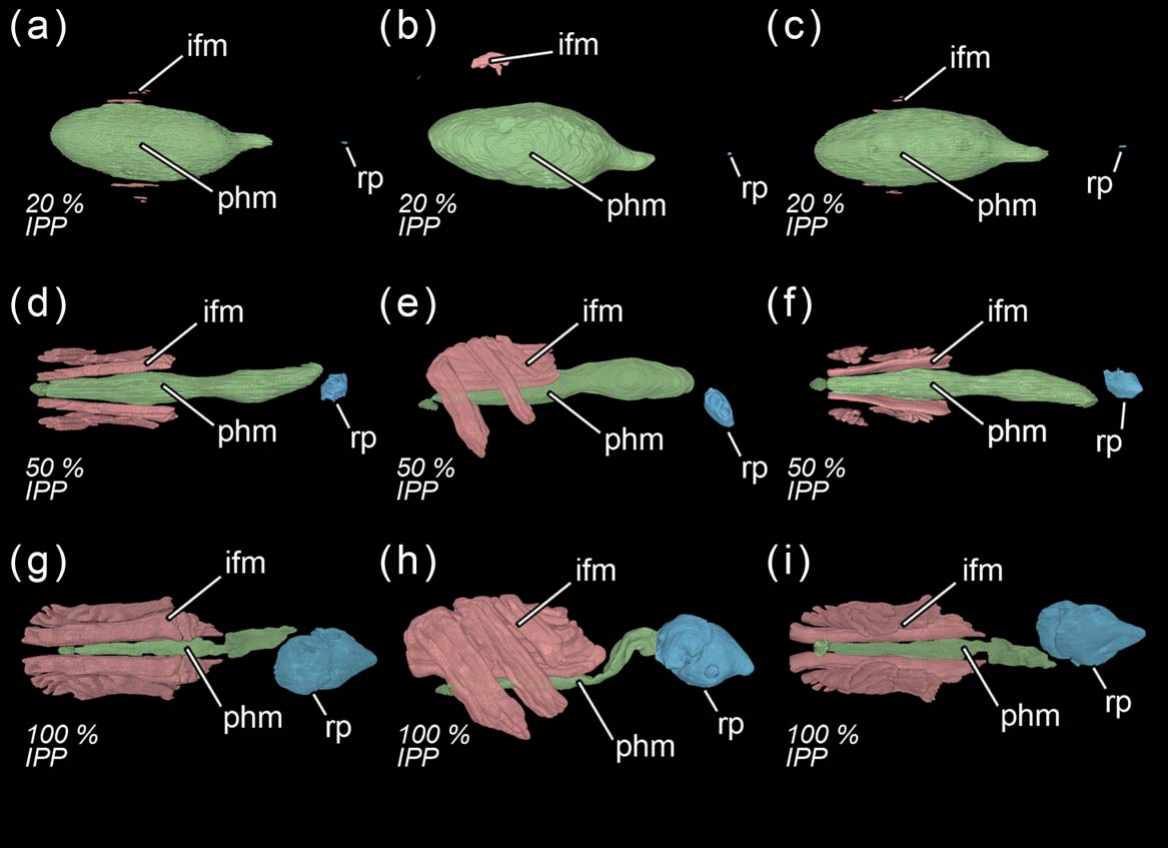
*Calliphora vicina*, volume renderings of the indirect flight muscles, the pre‐helicoidal region of the midgut and the rectal pouch at different times after pupariation (AP), reared at 24°C. The corresponding percentage of time of the total intra‐puparial period (IPP) is given in brackets after each time. (**a**) 48 hr AP (20% IPP), dorsal view. (**b**) 48 hr AP (20% IPP), left lateral view. (**c**) 48 hr AP (20 IPP), ventral view. (**d**) 120 hr AP (50% IPP), dorsal view. (**e**) 120 hr AP (50% IPP), left lateral view. (**f**) 120 hr AP (50% IPP), ventral view. (**g**) 240 hr AP (100% IPP), dorsal view. (**h**) 240 hr AP (100% IPP), left lateral view. (**i**) 240 hr AP (100% IPP), ventral view. ifm, indirect flight muscles; phm, pre‐helicoidal region of the midgut; rp, rectal pouch

The morphology of the pupa is very similar at 42 and 48 hr after pupariation (i.e., 17.5% and 20% of the total intra‐puparial period) (Figure 6a–f). Fat bodies and haemocytes are progressively migrating into the head, almost filling it (Figures 6a–f and 7a), and the antennae and external mouthparts are now discernible. Parts of the degenerating larval salivary glands are still visible (Figure 6f), whereas the adult salivary glands start to differentiate (Figure 6b,d). The optical nerve and the cornea, the latter visible as a more sclerotized layer, are present (Figure 6d,e), but the eye has not yet developed. The thoracic ganglion can be observed in the anterior part of the thorax (Figure 6a,c), and the adult tracheal system is developing with the formation of the pleural air sacs (Figure 6e). The pupal‐adult apolysis (i.e., the separation of the epidermal cells of the adult from the pupal cuticle) is ongoing, but not complete, in all three body regions (Figure 6a,b). The identification of the apolysing pupal cuticle in the virtual sections was confirmed by histological sections (Figure 7). The midgut is still closed and voluminous, occupying a significant portion of the thoracic diameter (Figures 4c, 6a–e, 8a, and 9a–c). Indeed, volume measurements of the adult midgut 48 hr after pupariation show a significant increase in comparison to the volume at 24 hr after pupariation, that is, when the gas bubble was present (Figure 8b). The adult hindgut starts to proliferate in the anterior part of the apoptotic larval hindgut (Figures 8c, 4c, and 6a,c); this is in accordance with Takashima et al. (2008), who identified a population of intestinal stem cells located in that area, called the hindgut proliferation zone. Depending on the speed of the proliferation, some specimens already have a partially developed rectal pouch in the posterior part of the adult hindgut (Figure 6c); although its average volume is virtually zero at this time (Figures 8c and 9a–c; Table 3). Furthermore, the histogenesis of the indirect flight muscles has started and small fibres of both dorso‐ventral and dorsal‐longitudinal muscles are present (Figures 6b, 8d, and 9a–c; Table 3). According to Fernandes, Bate, and Vijayraghavan (1991), myoblasts surround modified larval muscles and use them as templates for forming the dorsal longitudinal muscles (Figure 7b), whereas the dorsoventral muscles develop simultaneously without the aid of such templates.

**Table 3:** Volume measurements of the pre‐helicoidal midgut, the indirect flight muscles and the rectal pouch at different times from pupariation to adult emergence in *Calliphora vicina* reared at 24°C

Hours after pupariation	Developmental interval (as percentage of the total intra‐puparial period)	Relative volume of pre‐helicoidal midgut: average ± STD	Relative volume of pre‐helicoidal midgut: range	Relative volume of indirect flight muscles: average ± STD	Relative volume of indirect flight muscles: range	Relative volume of rectal pouch: average ± STD	Relative volume of rectal pouch: range
0	0%	0	0	0	0	0	0
24	10%	0.78 ± 0.3	0.64–1.32	0	0	0	0
48	20%	3.17 ±0.29	2.81–3.54	0.18 ± 0.08	0.11–0.28	0.0005 ± 0.0007	0–0.001
72	30%	2.63 ± 0.23	2.4–2.95	0.29 ± 0.08	0.15–0.37	0.01 ± 0.004	0.008–0.01
96	40%	2.15 ± 0.25	1.79–2.39	0.64 ± 0.22	0.42–0.94	0.03 ± 0.01	0.01–0.05
120	50%	1.52 ± 0.19	1.4–1.86	0.73 ± 0.17	0.57–0.95	0.07 ± 0.02	0.03–0.09
144	60%	1.18 ± 0.17	1.05–1.47	1.37 ± 0.13	1.23–1.55	0.09 ± 0.04	0.05–0.14
168	70%	0.76 ± 0.06	0.68–0.85	2.14 ± 0.34	1.76–2.69	0.09 ± 0.05	0.04–0.18
192	80%	0.51 ± 0.09	0.35–0.59	3.35 ± 0.22	3.13–3.69	0.61 ± 0.18	0.38–0.84
216	90%	0.42 ± 0.13	0.22–0.55	4.49 ± 0.57	3.88–5.33	1.52 ± 0.19	1.27–1.79
240	100%	0.35 ± 0.1	0.22–0.47	4.89 ± 0.52	4.48–5.78	1.55 ± 0.22	1.35–1.8

Relative volumes are given as a percentage of the total puparial volume.

### 3.3 Pharate adult

#### 3.3.1 30% Of the total intra‐puparial period

**Figure 10:**
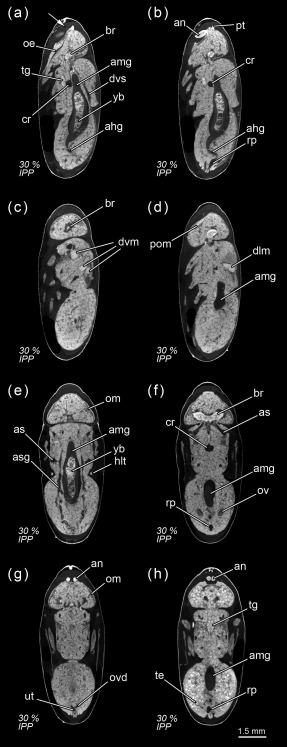
*Calliphora vicina*, micro‐CT‐based virtual sections of puparia 72 hr after pupariation (30% of the total intra‐puparial period, IPP), reared at 24°C. (**a**) medial sagittal section. (**b**) latero‐medial sagittal section. (**c**) lateral sagittal section. (**d**) lateral sagittal section. (**e**) dorsal horizontal section. (**f**) female, ventral horizontal section. (**g**) female, ventral horizontal section. (**h**) male, ventral horizontal section. ahg, adult hindgut; amg, adult midgut; an, antennae; as, air sac; asg, adult salivary gland; br, brain; cr, crop; dlm, dorsal longitudinal muscles; dvm, dorsoventral muscles; dvs, dorsal vessel; hlt, halters; oe, oesophagus; om, ommatidia; ov, ovaries; ovd, oviduct; pom, ptilino‐oesophagal muscle; pt, ptilinum; rp, rectal pouch; te, testes; tg, thoracic ganglion; ut, uterus; yb, yellow body

Seventy‐two hours after pupariation (i.e., 30% of the total intra‐puparial period), the pupal‐adult apolysis is complete over the entire body (Figure 10a–h) and the insect is therefore no longer a pupa but an adult, termed pharate adult as it is still within the puparium (Fraenkel & Bhaskaran, 1973; Hinton, 1946; Martín‐Vega et al., 2016). The midgut now occupies the anterior half of the abdomen and the hindgut is a continuous tube, connecting between the midgut and the rectal pouch (Figures 4d and 10b). The midgut is still voluminous (Figure 8b; Table 3), and it is now stretched anteriorly, being distinctly bottle shape (Figures 4d and 10a–b). The bottleneck or stretched region of the midgut is located in the same anterior section of the thorax where the thoracic ganglion is now positioned and where the crop and the crop‐duct are developing (Figures 4d and 10a,b). The oesophagus is also developing, although the pre‐ and post‐ganglionic sections still appear to be poorly developed and they are therefore difficult to segment by Avizo software (Figures 4d and 10a). The reproductive organs are also distinguishable at this time (Figure 10f–h). In the eyes, the ommatidia start to be discernible below the cornea (Figure 10e–h). Moreover, the first signs of the formation of the ptilinum (i.e., the eversible pouch above the base of the antennae used to push on and open the anterior end of the puparium in order for the adult fly to emerge) can also be seen from the onset of the pharate adult stage. A small ptilinal invagination (Figure 10b) and small fibres of the ptilino‐oesophageal muscle (Figure 10d) are already discernible at this stage.

#### 3.3.2 40% Of the total intra‐puparial period

**Figure 11:**
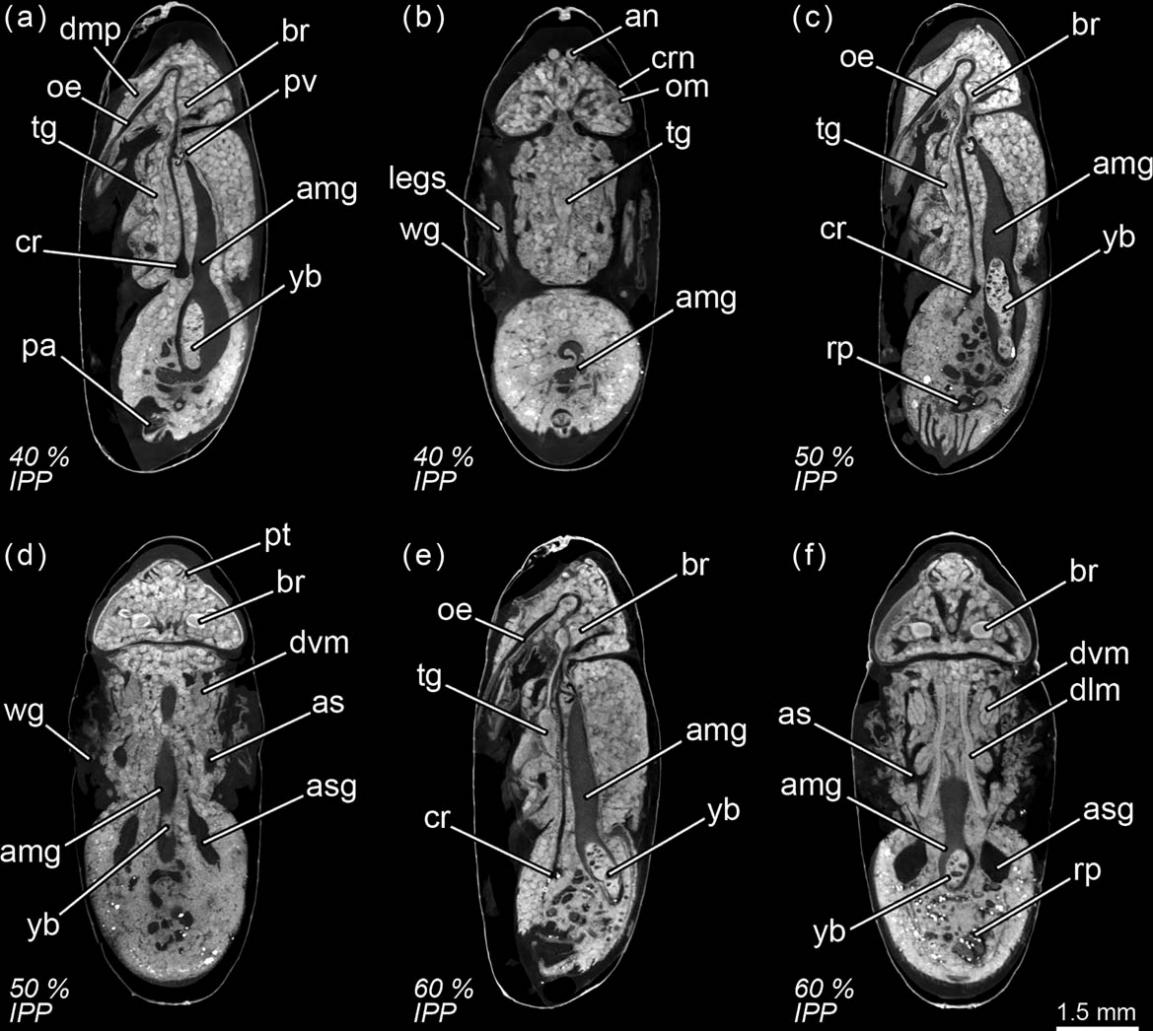
*Calliphora vicina*, micro‐CT‐based virtual sections of puparia at different times after pupariation (AP), reared at 24°C. The corresponding percentage of time of the total intra‐puparial period (IPP) is given in brackets after each time. (**a**) 96 hr AP (40% IPP), medial sagittal section. (**b**) 96 hr AP (40% IPP), ventral horizontal section. (**c**) 120 hr AP (50% IPP), medial sagittal section. (**d**) 120 hr AP (50% IPP), dorsal horizontal section. (**e**) 144 hr AP (60% IPP), medial sagittal section. (**f**) 144 hr AP (60% IPP), dorsal horizontal section. amg, adult midgut; an, antennae; as, air sac; asg, adult salivary gland; br, brain; cr, crop; crn, cornea; dlm, dorsal longitudinal muscles; dmp, dilator muscle of pharynx; dvm, dorsoventral muscles; legs, legs; oe, oesophagus; om, ommatidia; pa, penis apparatus; pt, ptilinum; pv, proventriculus; rp, rectal pouch; tg, thoracic ganglion; wg, wings; yb, yellow body

At 96 hr after pupariation (i.e., 40% of the total intra‐puparial period), the whole thoracic portion of the midgut is stretched (Figure 8a) as the crop‐duct grows and the crop is positioned in the posterior region of the thorax; the pre‐helicoidal region of the midgut thus acquires the shape of a long‐necked bottle (Figures 4e and 11a). The posterior end of the midgut grows and becomes helicoidal (Figures 4e and 11a–b). In the head, small fibres of the dilator muscle of the pharynx can be observed at this stage (Figure 11a).

#### 3.3.3 50% of the total intra‐puparial period

The crop reaches its final position in the anterior region of the abdomen 120 hr after pupariation (i.e., at 50% of the total intra‐puparial period), where it expands lateroventrally acquiring a flattened shape (Figures 4f and 11c). With the crop out of the thorax, the pre‐helicoidal region of the midgut becomes more cylindrical, decreasing the diameter of its abdominal section and increasing the diameter of the thoracic section (Figures 8a, 9d–f, and 11c). Both the helicoidal region of the midgut and the hindgut grow and become elongate (Figure 4f), developing their typical left‐right loops (Lengyel & Iwaki, 2002), thereby roughly acquiring the final shape of the alimentary canal of the adult (see Graham‐Smith, 1934). The adult salivary glands also appear to be fully developed at this time (Figure 11d).

#### 3.3.4 60% of the total intra‐puparial period

**Figure 12:**
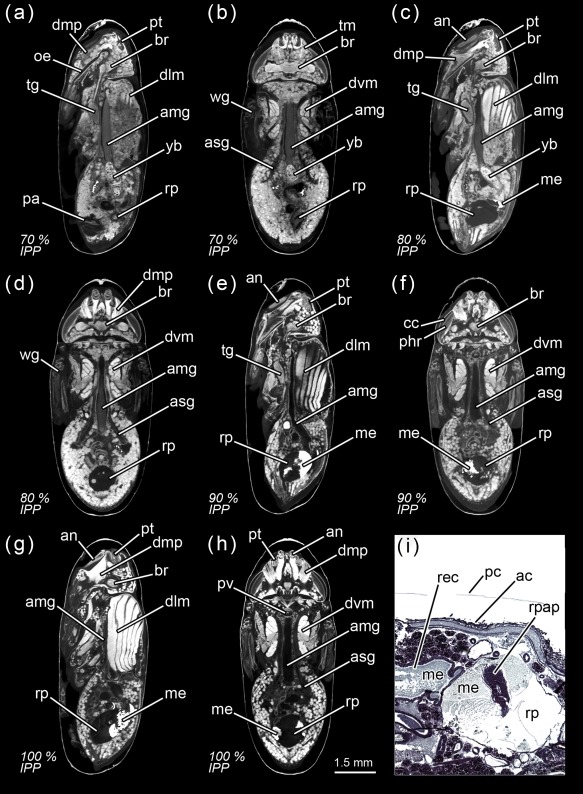
*Calliphora vicina*, micro‐CT‐based virtual sections of puparia at different times after pupariation (AP), reared at 24°C. The corresponding percentage of time of the total intra‐puparial period (IPP) is given in brackets after each time. (**a**) 168 hr AP (70% IPP), medial sagittal section. (**b**) 168 hr AP (70% IPP), ventral horizontal section. (**c**) 192 hr AP (80% IPP), medial sagittal section. (**d**) 192 hr AP (80% IPP), ventral horizontal section. (**e**) 216 hr AP (90% IPP), medial sagittal section. (**f**) 216 hr AP (90% IPP), ventral horizontal section. (**g**) 240 hr AP (100% IPP), medial sagittal section. (**h**) 240 hr AP (100% IPP), ventral sagittal section). (**i**) 240 hr AP (100% IPP), histological sagittal section of the abdomen showing the rectal pouch. ac, adult cuticle; amg, adult midgut; an, antennae; asg, adult salivary gland; br, brain; cc, crystalline cones; dlm, dorsal longitudinal muscles; dmp, dilator muscle of pharynx; dvm, dorsoventral muscles; me, meconium; oe, oesophagus; pa, penis apparatus; pc, pupal cuticle; phr, photoreceptor cells (and rhabdom); pt, ptilinum; pv, proventriculus; rec, rectum; rp, rectal pouch; rpap, rectal papillae; tg, thoracic ganglion; tm, tentorial muscles; wg, wings; yb, yellow body

**Figure 13:**
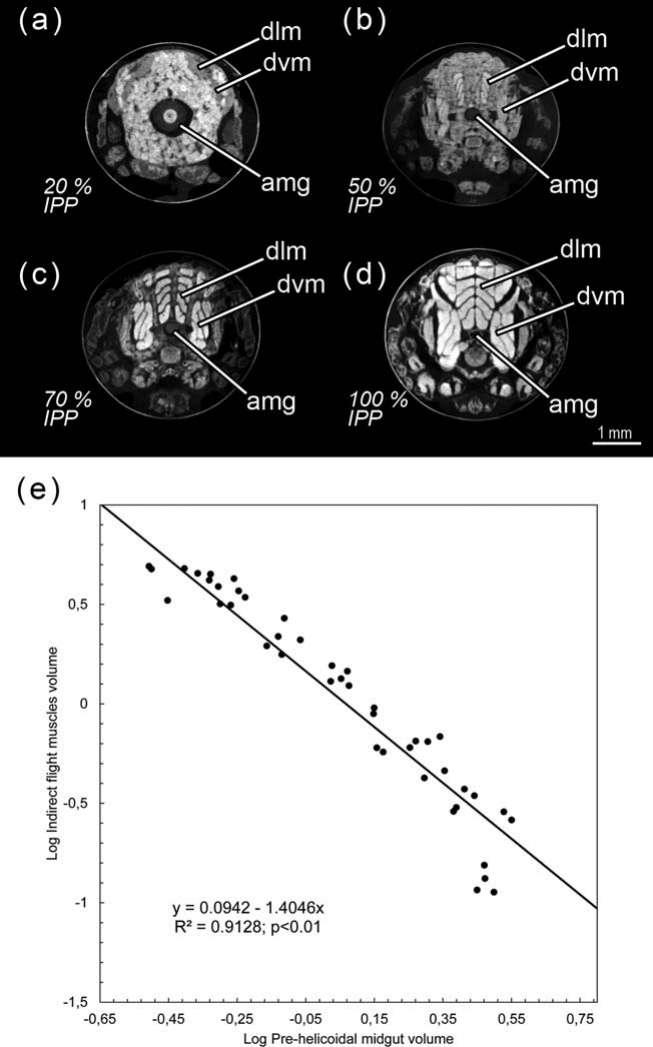
*Calliphora vicina*, micro‐CT‐based virtual cross sections of puparia and allometric relationship between the pre‐helicoidal region of the midgut and the indirect flight muscles during metamorphosis at different times after pupariation (AP), reared at 24°C. The corresponding percentage of time of the total intra‐puparial period (IPP) given in brackets. (**a**) 48 hr AP (20% IPP). (**b**) 120 hr AP (50% IPP). (**c**) 168 hr AP (70% IPP). (**d**) 240 hr AP (100% IPP). (**e**) Linear regression between the volume of the pre‐helicoidal region of the midgut and the indirect flight muscles during metamorphosis. amg, adult midgut; an, antennae; dlm, dorsal longitudinal muscles; dvm, dorsoventral muscles

**Figure 14:**
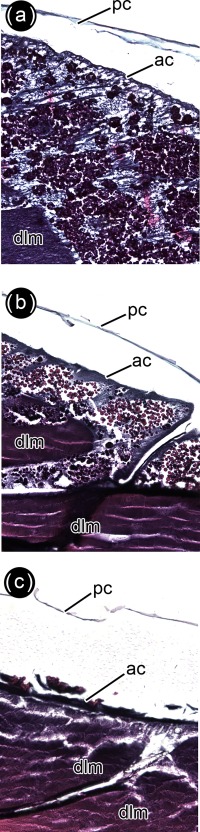
*Calliphora vicina*, histological sagittal sections of puparia reared at 24°C, showing the development of the indirect flight muscles at different times after pupariation (AP). The corresponding percentage of time of the total intra‐puparial period (IPP) is given in brackets after each time. (**a**) 120 hr AP (50% IPP). (**b**) 168 hr AP (70% IPP). (**c**) 240 hr AP (100% IPP). ac, adult cuticle; dlm, dorsal longitudinal muscles; pc, pupal cuticle

From 144 hr after pupariation (i.e., 60% of the total intra‐puparial period) until the end of the intra‐puparial period, the diameter and the volume of the pre‐helicoidal region of the midgut will decrease progressively (Figures 8a,b and 11e; Table 3) whereas the indirect flight muscles develop further (Figures 11f and 12a–h), increasing progressively in volume (Figure 8d) and occupying the majority of the thorax during the last intervals of the intra‐puparial period (Figure 12a–d). Indeed, the changes in the volume of the indirect flight muscles and the pre‐helicoidal region of the midgut during metamorphosis (Figure 8b,d) were significantly and negatively correlated, with a coefficient of determination (*R* ^2^) of 0.91 and a coefficient of correlation of −0.95 (Figure 13e). The negative value of −1.4 of the allometric coefficient (*k*) indicates a negative allometry, that is, the volume of the indirect flight muscles grows as the volume of the pre‐helicoidal region of the midgut decreases. The indirect flight muscles are attached to the thoracic cuticle—see Wisser and Nachtigall (1984) for details on the muscle insertion points—but during metamorphosis the fibres are mostly surrounded by haemocytes, fat bodies and fatty droplets (Figures 12a–e, 13a–c, and 14a,b)—see Crossley (1965) for more details. The indirect flight muscles will only attach to their corresponding insertion points in the thoracic cuticle at the end of the intra‐puparial period (Figures 12g, 13d, and 14c).

#### 3.3.5 70–80% Of the total intra‐puparial period

The final intervals of the intra‐puparial period are also marked by a significant increase in the volume of the rectal pouch (Figures 8c and 9g–i). There is a massive change between 168 and 192 hr after pupariation (i.e., 70% and 80% of the total intra‐puparial period, respectively), with an expansion of the lumen of the rectal pouch (Figure 12a–d) and the consequent increase in volume (Figure 8c; Table 3). At 192 hr after pupariation, part of the yellow body has been absorbed and the waste products have started to be transformed into the meconium, which is visualized in the virtual sections as an overstained (i.e., very bright) body in the rectum (Figure 12c).

#### 3.3.6 90–100% Of the total intra‐puparial period

At 216 and 240 hr after pupariation (i.e., 90% and 100% of the total intra‐puparial period, respectively), the yellow body has been completely absorbed and is no longer observable in the midgut, while the meconium fills the rectum and a considerable portion of the rectal pouch (Figure 12e–i). The rectal pouch goes through another significant increase in volume compared to the previous development interval (Figures 8c and 9g–i; Table 3).

In the head, the dilator muscle of the pharynx and the tentorial muscles have progressively developed from the small fibres present 96 hr after pupariation (Figure 11a) until its complete formation after 216–240 hr (Figure 12a–h). The scans also show a progressive development of the ommatidia from the onset of the pharate adult stage (Figure 10e–g, 11b,d,f, and 12b,d), with a clear differentiation of crystalline cones and rhabdom and photoreceptor cells arranged in two layers by 90% of the total intra‐puparial period, that is, 216 hr after pupariation (Figure 12e,h). A future study will explore in detail the development in the eyes (see second paragraph of Results). Previous studies have shown that the responses of the photoreceptor cells to light stimulus are certainly similar to those of the adult at this stage of development (Finell & Järvilehto, 1983). The ptilinal invagination reaches its final extension at the end of the intra‐puparial period (Figures 11d,f and 12a–h), when the pharate adult is ready to emerge from the puparium.

## 4 Discussion

### 4.1 The Metamorphosis of *Calliphora Vicina*

In cyclorrhaphous flies, the first signs of the degeneration of larval tissues are reported to appear immediately after pupariation (Levy & Bautz, 1985). This is supported by Cepeda‐Palacios and Scholl (2000) who showed how larval‐pupal apolysis also begins within 3 hr of pupariation in the sheep bot fly *Oestrus ovis* L. (Oestridae). However, this is in contrast to studies of other species such as *Sarcophaga bullata* Parker (Sarcophagidae) (Fraenkel & Bhaskaran, 1973) and *C. vicina* in which the apolysis was reported to start several hours after pupariation (see Table 4 for a compilation of the published data on apolyses timings of *C. vicina*). Nevertheless, our results show that the larval‐pupal apolysis must start shortly after pupariation in *C. vicina* as well, given that it is nearly complete in the thoracic region only 6 hr after pupariation (Figure 1d); that is, significantly earlier than hitherto described (Table 4). The timings of the apolyses in *C. vicina* determined by different studies are not fully concordant (Table 4), perhaps because of differences in the frequency of observations, with the data from Bautz (1971) being most in agreement with our observations. Determining when each apolysis is complete is fundamental to correctly establishing the duration of the different intra‐puparial stages in developmental studies (Fraenkel & Bhaskaran, 1973; Martín‐Vega et al., 2016) and our study considerably refines the timing of the apolysis events in *C. vicina* (Table 4). Furthermore, Cepeda‐Palacios and Scholl (2000) suggest interesting differences in the apolyses patterns, and in the relative duration of the intra‐puparial developmental stages among parasitic oestrid flies, as well as between them and other calyptrate muscoid families. We suggest that micro‐CT can be used for further explorations of potentially significant variations in the development of closely related Dipteran groups.

**Table 4:** Timetable for the start and completion of apolysis events during *Calliphora vicina* metamorphosis, in accordance with published data

Study	Temperature	Earliest record of start of larval‐pupal apolysis(hours after pupariation)	Earliest record of completion of larval‐pupal apolysis (hours after pupariation)	Earliest record of start of pupal‐adult apolysis (hours after pupariation)	Earliest record of completion of pupal‐adult‐apolysis (hours after pupariation)
Possompès (1953)	24–25°C	10	15	No data	Makes reference to a “second intra‐puparial moult, difficult to place in time”
Wolfe (1954)	24°C	No data	24–25	55–60	80
Pihan (1968)	25°C	12	24	48	72
Bautz (1971)	25°C	12	18	48	72
Current study	24°C	6	18	42	72

The actual start and completion times could be earlier due to non‐continuous sampling.

Among the morphological changes taking place during the metamorphosis of cyclorrhaphous flies, the full eversion of head, legs, and wings, which marks the transformation of the cryptocephalic pupa (Figure 3) into the phanerocephalic pupa (Figures 5 and 6), are indisputably the most striking, radical and extensive (Figure 2a). It has been suggested that the development of the gas bubble in the abdominal region may serve not only for maintaining a constant body volume within the puparium during a period of extensive histolysis (Langley & Ely, 1978) but also for aiding in the eversion of head, legs and wings by creating enough space between the pupa and the puparium, once the gas from the bubble has been released (Bainbridge & Bownes, 1981; Ždárek & Friedman, 1986). The role of the gas bubble in the eversion of those structures is described in another study (Hall et al., 2017), but the bubble must indeed be critical for this process, as *D. melanogaster* mutants which fail to release the gas, result in either crypto‐ or micro‐cephalic phenotypes (Rewitz et al., 2010). How the gas bubble forms is, nevertheless, still unknown. Ždárek and Friedman (1986) suggested that in the flesh fly *S. bullata*, it is formed by inflation of the midgut 15–20 hr after pupariation. However, in *C. vicina*, the gas bubble appears 3–4 hr after pupariation (Figures 1 and 2) and its volume increases before the adult midgut inflates (Figure 3c–d). Langley and Ely (1978) speculated that in the tsetse fly *Glossina morsitans* Westwood (Glossinidae), the bubble might be secreted from one of the tracheal trunks. Bainbridge and Bownes (1981) also suggested that there might be connections between the bubble and the tracheal system in dissected prepupae of *D. melanogaster*. Our scans show for the first time the connection of the bubble with one of the main tracheal trunks (Figure 1h–i) which, as well as the X‐ray images (Figure 2a) revealing the placement of the bubble close to one of the main tracheal trunks—or two bubbles placed close to each main tracheal trunk in some individuals—strongly supports the hypothesis of a tracheal origin. Further studies are needed to determine whether the bubble is formed by simple air intake through the tracheal system or if it is developed by a cavitation‐like process under negative pressure as a consequence of the rapid water loss recorded during the prepupal and pupal stages of cyclorrhaphous flies (Gilby & Rumbo, 1980; Zajac & Amendt, 2012).

Interestingly, once the gas bubble has been released and the head everts, the lumen of the adult midgut expands as if to occupy the space left vacant by the bubble (Figures 5a,d and 8a–b). Langley and Ely (1978) suggest that the development of the gas bubble may act as a compenzation mechanism enabling the insect to decrease its mass without decreasing its volume within the puparium during the prepupal and the cryptocephalic pupal stages, and they found a significant correlation between the bubble volume and the puparial weight loss in *G. morsitans*. Considering that there is a highly significant weight loss between the cryptocephalic and the phanerocephalic pupal stages in *C. vicina* (Zajac & Amendt, 2012), our results suggest that the adult midgut may replace the gas bubble, while maintaining the body volume constant within the puparium during the phanerocephalic pupal stage. Moreover, the significant negative correlation between the changes in volume of the indirect flight muscles and the pre‐helicoidal region of the midgut (Figure 13) suggest that the development of both organ systems might be somewhat connected, with the indirect flight muscles progressively replacing the midgut as the largest organ within the thorax toward the end of the intra‐puparial period (Figure 8b,d). There is evidence from other studies to support the hypothesis that organ growth in insects might be at least partly regulated by feedback between growing organs to modulate final sizes, including negative regulation (Stern & Emlen, 1999). Our results might be the first indirect evidence supporting this hypothesis in the metamorphosis of cyclorrhaphous flies. Nevertheless, the genetic architecture of insect body size appears to be complex, involving a large section of the genome (Carreira, Mensch, & Fanara, 2009). A recent study (Zajac, Amendt, Horres, Verhoff, & Zehner, 2015) identified differentially expressed genetic markers at specific points of the intra‐puparial development of *C. vicina*, with promising results not only for forensic research but also for developmental biology. Future investigations, ideally integrating both molecular and morphological analyses, may shed more light on the identification and relevance of the genes involved in the regulation of the size of different organ systems during metamorphosis.

### 4.2 Micro‐CT as a tool for intra‐puparial development studies

The use of micro‐CT has a series of practical advantages over traditional histological studies as already highlighted by several authors (Carbayo & Lenihan, 2016; Lowe et al., 2013; Smith et al., 2016; Richards et al., 2012). Among the most important advantages, micro‐CT is a significantly less time‐consuming process and a much less invasive analysis of the sample which, subsequently, can be virtually dissected in any plane. Traditional histology is particularly challenging in the case of cyclorrhaphous fly puparia because of the profusion of fat bodies and fatty droplets (Davies & Harvey, 2013) resulting from the histolysis of larval tissues (Crossley, 1965), as well as the hardness and impermeability of the puparium, which makes the infiltration of the internal tissues with the embedding material difficult. Davies and Harvey (2013) tested different fixation and preservation protocols but in every case they obtained frequently fragmented sections, thus losing significant parts of the sample tissues. These drawbacks of histological studies can severely limit the morphological analyses and preclude obtaining quantitative data. In fact, those limitations can be extended to any method involving a sectioning of the sample, such as immunohistochemistry, transmission electron microscopy or serial block‐face scanning electron microscopy, which moreover is limited to very small size samples (see Friedrich et al., 2014 for a critical review on different imaging methods). Conversely, whereas other techniques such as autofluorescence microscopy can be used to document developmental processes *in toto* in those insects with a transparent pupal cuticle (Saltin, Haug, & Haug, 2016), this is not the case for cyclorrhaphous flies, where the opaque puparium certainly impedes the visualization of the morphological changes taking place inside. Micro‐CT is in this sense a powerful tool for quantitative and qualitative analyses *in toto* of the internal morphology of intra‐puparial specimens of cyclorrhaphous dipterans, with the additional advantage of not dissecting samples which might be legal evidence in specific situations if we consider the forensic relevance of species like *C. vicina* (Richards et al., 2012).

We must emphasise, however, that micro‐CT should not be considered a complete substitute for other imaging methods for morphological analyses, but should rather be a complementary technique. As suggested by Saltin et al. (2016), the combination of the appropriate method and the right organism can provide new insights into specific questions on specific developmental processes. With the current available resolution, micro‐CT scans cannot document changes at the cellular level (Richards et al., 2012). For example, monitoring the histolysis and histogenesis of individual hypodermal muscles at the cellular level during metamorphosis, where certain larval muscles transform into adult ones, requires the higher resolution power that can be delivered by other methods, like traditional histology (Crossley, 1965; Zajac & Amendt, 2012) or transmission electron microscopy (Takashima et al., 2008). Conversely, the muscles, as well as other tissues, require staining by both techniques and therefore each specimen can be measured only once during its development, losing information on potential individual variation in growth (Cock, 1966). The use of iodine in the micro‐CT staining used here can result in overstained structures like the apoptotic larval salivary glands in the current scans (Figure 3c,d,g), probably due to a reaction between the iodine and the products from phosphatase hydrolysis taking place in cellular cytoplasm at that point (Levy & Bautz, 1985). There are a variety of staining solutions which can also produce high differential tissue contrast for X‐ray imaging of tissues (Metscher, 2009). Further research on the suitability of different staining methods for specific tissues and optimal staining times (Smith et al., 2016) may overcome these drawbacks in the near future.

Nevertheless, the current research supports and significantly extends the previous work of Richards et al. (2012) and Lowe et al. (2013), further demonstrating the value of micro‐CT as a powerful tool for monitoring and understanding the massive morphological changes taking place during the metamorphosis of holometabolous insects. For the first time, the major morphological changes in key internal structures during the pupal stage have been imaged and documented *in toto* on a cyclorrhaphous fly species, whereas the internal morphological changes taking place in the pharate adult have been revealed at a finer temporal and spatial resolution than anything hitherto published (Richards et al., 2012). This refinement has allowed for the correction of some misinterpretations from previous studies, like the significantly later development of key structures like the ptilinum, the optic nerve or the rectal pouch suggested by Richards et al. (2012), which however are already present at the onset of the pharate adult stage or even earlier (Table 1). Also, the use of micro‐CT enabled delimiting the start and completion of the apolysis events with higher precision (Table 4), which is crucial for a correct determination of the duration of the different developmental stages (Martín‐Vega et al., 2016). Furthermore, we have successfully applied the method described by Lowe et al. (2013) for performing quantitative ontogenic analyses in studies of insect metamorphosis. The current study provides the first quantitative analysis of the development of selected organ systems in cyclorrhaphous dipterans, introducing exciting new dimensions into developmental and comparative studies. In the specific case of forensically relevant species, like *C. vicina*, quantitative analyses of selected structures may provide an additional measure of insect age for minimum post‐mortem interval estimations. We must highlight that the use of a software tool for automatic segmentation—the Avizo's “Magic wand” tool used here—required in every case reviewing the virtual slices and manual correction by adding or removing areas where needed. For example, the indirect flight muscles usually showed contrast levels similar to the adjacent fat bodies, resulting in the inclusion of the latter in the automatic segmentation. However, this approach undoubtedly saves a considerable amount of time in comparison to a complete segmentation by hand when large data sets need to be analyzed. Different image analysis methods for micro‐CT studies of insect internal morphology have been recently explored (e.g., Lowe et al., 2013; Simonsen & Kitching, 2014; Smith et al., 2016). Therefore, as with the choice of the imaging technique, certain image analysis methods will be appropriate for certain questions.

## Author's contributions

DMV, TJS and MJRH conceived and designed the study. DMV conducted the experiments, and analyzed the data. DMV, TJS and MJRH all contributed to the interpretation of the data. DMV wrote the manuscript, which was read, corrected and approved by all authors.
